# Amelogenin-inspired peptide, calcium phosphate solution, fluoride and their synergistic effect on enamel biomimetic remineralization: an in vitro pH-cycling model

**DOI:** 10.1186/s12903-024-04008-z

**Published:** 2024-02-27

**Authors:** Aliaa H. Sakr, Mohammed Salah Nassif, Dalia I. El-Korashy

**Affiliations:** Dental Biomaterials, Biomaterials Department, Faculty of Dentistry, Ain-Shams University, Organization of African unity street, El-Qobba Bridge, El-Weili, Cairo, Egypt

**Keywords:** Peptide, Fluoride, Biomimetic, X-ray diffraction, Nanoindentation, Raman spectroscopy

## Abstract

**Background:**

Several methods were introduced for enamel biomimetic remineralization that utilize a biomimetic analogue to interact and absorb bioavailable calcium and phosphate ions and induce crystal nucleation on demineralized enamel. Amelogenin is the most predominant enamel matrix protein that is involved in enamel biomineralization. It plays a major role in developing the enamel’s hierarchical microstructure. Therefore, this study was conducted to evaluate the ability of an amelogenin-inspired peptide to promote the remineralization potential of fluoride and a supersaturated calcium phosphate solution in treating artificially induced enamel carious lesions under pH-cycling regimen.

**Methods:**

Fifty enamel slices were prepared with a window (4*4 mm^2^ ) on the surface. Five samples were set as control healthy enamel and 45 samples were subjected to demineralization for 3 days. Another 5 samples were set as control demineralized enamel and 40 enamel samples were assigned into 8 experimental groups (n=5) (P/I, P/II, P/III, P/AS, NP/I, NP/II, NP/III and NP/AS) according to peptide treatment (peptide P or non-peptide NP) and remineralizing solution used (I; calcium phosphate solution, II; calcium phosphate fluoride solution, III; fluoride solution and AS; artificial saliva). Samples were then subjected to demineralization/remineralization cycles for 9 days. Samples in all experimental groups were evaluated using Raman spectroscopy for mineral content recovery percentage, microhardness and nanoindentation as healthy, demineralized enamel and after pH-cycling. Data were statistically analysed using two-way repeated measures Anova followed by Bonferroni-corrected post hoc test for pairwise multiple comparisons between groups. Statistical significance was set at p= 0.05. Additionally, XRD, FESEM and EDXS were used for crystal orientation, surface morphology and elemental analysis after pH-cycling.

**Results:**

Nanocrystals clumped in a directional manner were detected in peptide-treated groups. P/II showed the highest significant mean values in mineral content recovery (63.31%), microhardness (268.81±6.52 VHN), elastic modulus (88.74±2.71 GPa), nanohardness (3.08±0.59 GPa) and the best crystal orientation with I_002_/_I300_ (1.87±0.08).

**Conclusion:**

Despite pH changes, the tested peptide was capable of remineralizing enamel with ordered crystals. Moreover, the supplementary use of calcium phosphate fluoride solution with peptide granted an enhancement in enamel mechanical properties after remineralization.

## Background

Dental caries is a major prevalent chronic disease as it affects a large sector of the population worldwide [1, 2]. Demineralization is considered the first step in forming dental caries that ends with losing structural integrity [3]. However, remineralization cycles always interfere and alternate with demineralization cycles, diverting this process away from cavitation [4]. Accordingly, modern dentistry focused on replacing traditional restorative methods with more effective modalities that treat non-cavitated demineralized enamel non-invasively through remineralization [2, 4–6].

Several remineralizing agents and techniques have been researched with significant positive results and were translated to be clinically applied [7, 8]. Fluoride-based treatment is still the standard therapy for remineralizing enamel carious lesions. Fluoride plays a major role in interrupting enamel dissolution and degradation as it hastens the formation of new larger fluorapatite crystals. Several studies suggested a dose-response characteristic for fluoride [9–12]. In 2019, a randomized clinical trial observed a significant increase in fluoride levels in plaque and saliva after using 5000 ppm fluoride dentifrices in comparison to another one containing only 1000 ppm of fluoride [10]. Deductively, low fluoride dosage needs a complementary treatment to aid fluoride binding and its subsequent release into saliva over time.

Currently, the net remineralization results from using topical fluoride application are limited by the bioavailability of calcium (Ca^2+^) and phosphate (PO_4_^3-^)ions. The addition of extrinsic sources of Ca^2+^ and PO_4_^3-^ ions in remineralization protocols can augment fluoride-mediated remineralization by increasing ions diffusion gradients [6]. Normally, the Ca/P molar ratio in the plaque is ~ 0.3. However, for an ideal enamel remineralization, a Ca/P molar ratio of 1.6 is needed to provide an equal rate of supersaturation. Therefore, a supplemental source for ions is required to enhance enamel remineralization [13].

In recent years, efforts have been directed toward understanding tooth structure's physicochemical and biological mineralization mechanisms. Enamel formation is a typical biomineralization process that requires synergistic interaction of both organic and inorganic components [14]. Therefore, based on the principles of biomineralization, efforts in tissue engineering were directed to develop biomimetic remineralization systems that demonstrate promising potentials for the regeneration of enamel’s hierarchical microstructure and its mechanical performance [15, 16].

Biomimetic remineralization systems should fulfil specific criteria to ensure sufficient concentration of Ca^2+^ and PO_4_^3-^ ions at the sites to be remineralized as well as the presence of biomimetic analogues that are able to strongly absorb both Ca^2+^ and PO_4_^3-^ ions and subsequently prevent demineralization. Several short-functioning peptides were reviewed in the literature; for example, peptides derived from dentin phosphoprotein (DPP) and amelogenin-based polypeptides showed promising remineralization [17–21].

Amelogenin is considered the major protein in enamel matrix that plays a principal role in its formation. The chemistry of amelogenin enables it to assemble itself into spherical nanospheres, oligomers and nanoribbons under different in vitro conditions; thus, it can promote crystal organization [2, 6, 21]. Several research optimized the use of amelogenin as a preventive strategy on etched enamel where leucine-rich amelogenin and amelogenin-inspired peptides showed promising capabilities in growing enamel-like apatite crystals and repairing its mechanical strength [19, 21, 22]. Mukherjee et al. have designed a medium- length amelogenin inspired peptide preserving parts of the C and N- terminals of amelogenin. This peptide succeeded in remineralizing enamel with highly oriented crystals and increasing its mechanical strength [21, 23].

Previous studies revealed that several factors may affect the phase formation and morphology of calcium phosphate crystals using biomimetic remineralization systems. The degree of supersaturation of the remineralizing solution, pH, temperature, and the presence of certain ions, such as fluoride, have been reported to affect the crystal morphology dramatically [11].

Hence, the objective of this study was to evaluate the ability of this peptide to work synergistically with supersaturated calcium phosphate solution and /or fluoride and remineralize enamel under pH-cycling. Furthermore, to evaluate the quality of the newly formed apatite layer (composition, orientation), as well as the enamel surface mechanical properties.

## Materials and methods

### Sample size calculation

Sample size for the study was calculated using G. Power program 3.19.2 software according to a pilot study based on surface microhardness and nanoindentation tests. A sample size of 5 samples per group achieved a power of 93% using F-test for one-way Anova with level of significance of 0.05.

### Teeth selection and sample preparation

Thirty-two sound human third molars were collected with age ranging from 25 to 40 years with patients’ informed consent. Molars were selected with no visible white spot lesions or discoloration, rinsed with 70% ethanol, then sonicated in distilled deionized water (DDW) for 20 minutes and stored in 0.05% thymol at 4°C until use within a maximum of one month [21, 24]. For enamel sample preparation, 3 mm thick slices were then longitudinally sectioned in a mesiodistal direction from the buccal and lingual surfaces using an Isomet saw (Isomet 4000, Buehler, Lake Bluff, IL, USA) at a speed of 2500 rpm producing 64 enamel slices.

The enamel surface of each tooth slice was then polished to ground flat with wet silicon carbide papers in a sequential grit series of 600, 800, 1200, 2000, 2500, 15 s each, to create standardized smooth surfaces [25]. Enamel slices were then rinsed and sonicated in a DDW bath for 5 min to remove any residual abrasives and finally stored in DDW at 4°C for further use [21, 26].

**Fig. 1:**
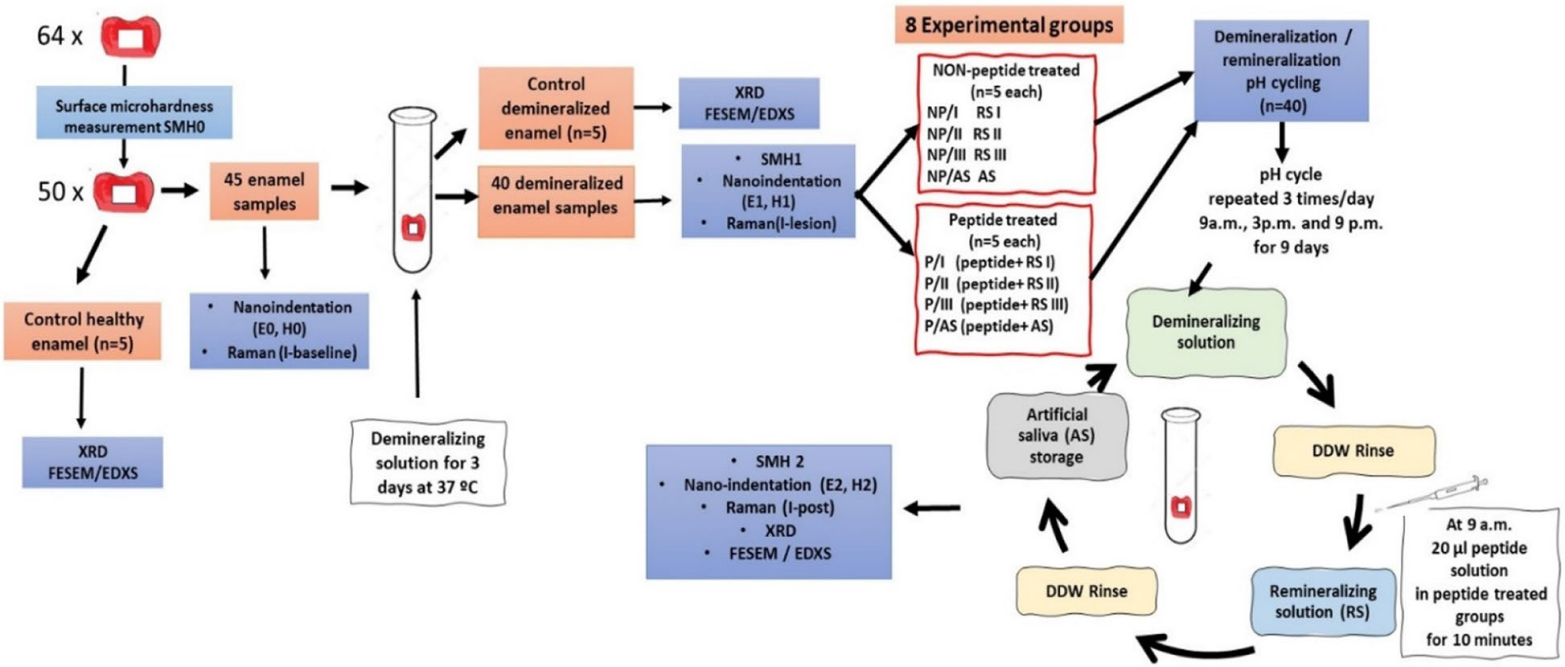
Experimental design

The surface of each enamel slice was coated with 2 layers of acid-resistant nail varnish (Yolo cosmetics), leaving a small window (4×4mm^2^) for demineralization and remineralization treatments. A shallow vertical groove was performed using a low-speed disc on the under surface (dentin surface) of each slice in the middle of the window, then the surface was sealed with nail varnish [19]. Enamel surface microhardness (SMH) was measured for all samples using Vickers hardness tester (Nexus 4000 TM, INNOVATEST, model no. 45.3 I, Netherlands) with loading force of 100 g and loading time of 20 s and the average value of three readings was calculated for each sample [27, 28]. For standardization, a total of 50 samples with recorded microhardness values ranging from 250-300 VHN were selected and set as the baseline surface microhardness (SMH0). Fourteen enamel samples were discarded, being out of the set VHN range. Five enamel samples were kept to be analyzed as control healthy enamel for scanning the surface morphology and X-ray diffraction analysis (Fig. 1).

### Preparation of the demineralized enamel model

To create artificial initial carious lesions in 45 enamel samples, each sample was immersed individually in 16 ml of demineralizing solution for 3 days at 37 °C [24, 29–31]. The demineralizing solution was prepared with a composition of 50 mM acetic acid (pH 4.5), 2.2 mM Ca(NO_3_)^2^, 2.2 mM KH_2_PO_4_, 5.0 mM NaN_3_, and 0.5 ppm NaF and the pH was adjusted to 4.5 with NaOH [24, 29, 30, 32]. The demineralizing solution was refreshed daily and after 3 days, enamel samples were rinsed and ultrasonically cleaned in DDW 3 times (5 min each) to terminate the demineralization process [31]. Another 5 samples were assigned as the control demineralized group for scanning the enamel surface and X-ray diffraction test (Fig. 1). The surface microhardness for the remaining 40 demineralized samples was then measured as previously described and SMH1 was recorded for each sample.

### Preparation of the peptide and remineralizing solutions

A Fluorescein isothiocyanate (FITC) labelled peptide, inspired by amelogenin, with amino acids sequence of (MPLPSYEVLTPLKWPSTDKTKREEVD) was synthesized by LifeTein, LLC, New Jersey, USA using solid phase peptide preparation method. The molecular mass of the peptide and purity were determined (Table 1), by the company prior to shipment, using Mass Spectrometry and High-performance liquid chromatography, respectively. Additionally, the peptide net charge at pH 7, isoelectric point IP and average hydrophilicity were calculated (Table 1).

**Table 1:** Molecular mass, purity, charge, isoelectric point IP and average hydrophilicity of the peptide

Mass (Da)	Purity %	Net Charge	IP	Average hydrophilicity
3643.04	96.05	-1.99	4.36	42

For preparation of the peptide stock solution, 2 mg of the peptide powder were weighed and dissolved in 10 ml DDW, then centrifuged for 2 minutes at 8000 rpm. A series of daily working aliquots of the peptide was prepared by dividing the peptide stock solution into 10 Eppendorf tubes with 1ml/ tube, followed by shaking the tubes for 4 h at 37°C in a water bath shaker. Aliquots were kept at -80°C for 12 hours until lyophilized with a final concentration of 0.2 mg (200 μg) per tube, then stored at 4°C. On the intended day of application, each aliquot was redissolved again in 1 ml of DDW to get a final concentration of 0.2 mg/ml per tube [21, 33].

Three remineralizing solutions (RS) were prepared with the following compositions: solution I; 4.80 mM CaCl_2_ and 2.89 mM Na_2_HPO_4_ in 50 mM Tris-Buffer Solution (TBS), solution II; 4.80 mM CaCl_2_, 2.89 mM Na_2_HPO_4_ and 1100 ppm NaF in 50 mM TBS and solution III; 1100 ppm NaF in 50 mM TBS with a pH of 7.2 (close to salivary pH) [19]. Artificial saliva (AS) was also prepared containing (1.2 mM CaCl_2_·2H_2_O, 50 mM HEPES buffer, 0.7 mM KH_2_PO_4_,16 mM KCl, 4.5 mM NH_4_Cl, 0.2 mM MgCl_2_·6H_2_O, and 1ppm F) adjusted at pH 7.2 [21, 33].

### Samples’ grouping and demineralization-remineralization (pH-cycling) protocol

As shown in Fig. 1, 40 enamel samples were randomly assigned into two major groups; peptide-treated (P) and non-peptide treated (NP). Then, each group was subdivided into 4 subgroups (n= 5) according to the remineralizing solution used (P/I, P/II, P/III, P/AS) and (NP/I, NP/II, NP/III, NP/AS). Enamel samples were exposed to a regimen of controlled pH-cycling over a period of 9 days. During each 24-hour period, all samples were subjected to pH-cycling 3 times, at 9 am, 3 pm and 9 pm. During every cycle, each sample was immersed individually in 11ml of demineralizing solution for 20 min, rinsed with DDW then immersed again in another 11ml of the remineralizing solution assigned for its group for another 20 minutes. After demineralization / remineralization cycle, samples were rinsed again with DDW and finally stored in artificial saliva until the next cycle.

At 9 am each day, after demineralization and rinsing with DDW, 20 μL (0.004 mg) of the peptide solution were dropped over the exposed window of enamel samples in peptide-treated groups (n=20) and left for 10 minutes to ensure complete drying of the enamel surface before immersion in the remineralizing solution [21, 33]. In group P/AS and NP/AS, artificial saliva was considered the remineralizing solution.

### Spontaneous mineralization testing

Transmission electron microscope (TEM) with selected area electron diffraction (SAED), ( Jeol, JEM-2100 PLUS, accelerating voltage, 200 kV ) was used to evaluate the effect of the tested peptide on Ca-P mineralization in vitro using different remineralizing solutions. Four stock solutions (A, B, C and D), 20 μL each, were prepared as following: solution A; 10 μL of remineralizing solution I and 10 μL of artificial saliva, solution B; 10 μL of remineralizing solution II and 10 μL of artificial saliva, solution C; 10 μL of remineralizing solution III and 10 μL of artificial saliva and solution D; 20 μL of the prepared artificial saliva.

Eight carbon film supported copper grids with standard thickness and size 200 mesh were used, 4 grids representing peptide-treated groups, and the other 4 grids for non-peptide treated groups. In peptide-treated groups, 5 μL of the peptide solution were first pipetted onto each of the 4 grids and blotted with filter paper. Then grids were treated with 10 μL of solutions (A, B, C and D) to represent groups (P/I, P/II, P/III and P/AS), respectively. In non-peptide treated groups, grids were directly treated with the prepared solutions (A, B, C and D) designated for (NP/I, NP/II, NP/III and NP/AS), respectively. All grids were blotted and scanned after 20 minutes then after 24 hours [21].

### Binding capacity of FITC-labelled peptide to enamel surface

Confocal laser scanning microscopy (CLSM, Olympus, Tokyo, Japan) at an excitation wavelength of 488 nm, was used to evaluate the binding capacity of the FITC-labelled peptide to healthy and demineralized enamel. First, enamel samples of control healthy and demineralized enamel groups (n=5) were visualized using CLSM and images were set as negative control. Then, 20 μl of the peptide solution were dropped on the surface of the enamel window of all samples, left undisturbed for 10 minutes at room temperature, rinsed three times with DDW, air dried and visualized again using CLSM [29, 32]. The Binding capacity of FITC-labelled peptide was measured as a function of the percentage of area covered with fluorescent peptide in relation to the total area scanned. CLSM images were analyzed using Image J (1.41a, NIH, USA).

### Structural characterization of remineralized enamel

#### X-ray diffraction (XRD) analysis

XRD; Panalytical X’Pert Pro diffractometer (Malvern Panalytical Ltd, Malvern, UK) was used to characterize the newly formed mineral crystals and its preferential orientation on the surface of each sample (n=5) in 8 remineralizing experimental groups and control healthy and demineralized enamel groups. XRD equipped with a secondary monochromator, utilizing (Cu) K_α_ radiation with λ=1.542 Å, operating at 45 kV and 35 M.A with a scanning speed of 0.04/sec was used. Diffraction peaks between 2θ = 5° and 60° and their corresponding spacing (d) were obtained and compared to (ICDD) files 09-0432 of hydroxyapatite (HAP) [21, 33]. The diffraction intensity of peaks at ~25.9° and ~32.9° corresponding to (002) and (300) planes, respectively, were measured for each sample using OriginPro software (OriginPro 2018, OriginLab, Northampton, MA, USA). Then, the ratio of the diffraction intensity of peak (002) to (300) (I_002_:I_300_) was calculated for each sample and used to evaluate the degree of orientation along the c-axis in each of the 8 experimental groups in comparison to both control healthy and demineralized enamel [21, 33, 34].

#### Raman micro-spectroscopic analysis

High-resolution confocal Laser Raman microspectroscopy (Senterra, Bruker Optics, Germany) was used for molecular assessment of the newly formed surface mineral layer on enamel after remineralization in different remineralizing groups (n=5). It was operated using a 785 nm near-infrared laser diode at power of 100 mW and spectra were measured over the range of 45–4500 cm^−1^. Each sample was scanned 3 times as healthy, demineralized and remineralized enamel (Fig*.* 1). After gaining spectral data, the exact position of (PO_4_^3-^) was detected and its Raman relative intensity were calculated using OriginPro software. To quantify the surface mineral content recovery of remineralized enamel, Raman relative intensity of (PO_4_^3-^) was used as enamel mineral content indicator and the surface mineral content recovery of remineralized enamel was calculated as intensity change ratio % (ICR%) according to the following formula (Equation 1) [35, 36]:

1 $$\%\textrm{ICR}=\left[\left(\textrm{I}-\textrm{Post}/\textrm{I}-\textrm{Baseline}\right)-\left(\textrm{I}-\textrm{Lesion}/\textrm{I}-\textrm{Baseline}\right)\right]\times 100\%.$$

Where (ICR%) : intensity change ratio %, I-Baseline: Raman relative intensity at ~ 960cm^-1^ of healthy enamel, I-Lesion: Raman relative intensity at ~ 960 cm^-1^of demineralized enamel and I-Post: Raman relative intensity at ~ 960 cm^-1^ of remineralized enamel.

#### Field emission scanning electron microscopy / Energy-dispersive X-ray spectrometry

Before scanning, a chisel was carefully used to fracture the samples of the different experimental groups, control healthy and demineralized enamel (n=5) along the groove in the middle of the dentin under surface of each sample. Each sample was fractured into 2 equal parts, one part for surface scanning and the other part was used for cross-sectional scanning. The surfaces of both parts were gold sputtered with a current of 15 mA for 3 minutes. Field emission scanning electron microscope FESEM ( LEO SUPRA 55, Carl Zeiss, Germany) operated at 8.00 kV in high vacuum conditions was used for morphological analysis. Samples were first dried and desiccated prior to placement in the SEM chamber and imaged at 3000X and 10000X magnifications. Energy-dispersive X-ray spectrometry EDXS (Neoscope JCM 6000 plus Joel benchtop SEM, Nikon, Japan) operated at 15 kV with a probe current 7.47500 nA was used for elemental analysis of enamel surfaces. X-ray spectra were acquired, and analysis was performed in terms of atomic and mass percentage of elements, specifically O, C, Ca, P, and F.

### Mechanical properties measurement

#### Surface Microhardness

Evaluation of the mechanical properties of remineralized enamel surface in different remineralizing groups was performed before FESEM/EDXS test. Microhardness of enamel surfaces in the 8 experimental groups (n=5) after pH-cycling (SMH2) were recorded following the same specifications previously mentioned. The surface microhardness recovery ratio (%SMHRR) for each sample was then calculated based on the following formula (Equation 2) [24, 29, 32, 35, 37]:

2 $$\%\textrm{SMHRR}=\left(\textrm{SMH}2-\textrm{SMH}1\right)/\left(\textrm{SMH}0-\textrm{SMH}1\right)\times 100$$

Where SMHRR%: surface microhardness recovery ratio, SMH0: surface microhardness of healthy enamel, SMH1: surface microhardness of demineralized enamel, SMH2: surface microhardness of remineralized enamel

#### Nanoindentation

The surface nanohardness H and elastic modulus E of enamel samples (n=5) for the 8 different experimental groups were measured for healthy, demineralized and remineralized enamel (Fig*.* 1) using Nanoindentation test with continuous stiffness method (CSM). Nanoindenter-XP, operated with Agilent-Nanosuit software, equipped with a Berkovich diamond indentation tip (less than 100 nm curvature), was used to make 25 indentations on each sample [21, 33]. The test was conducted using the following parameters: vibration frequency of 45 Hz, amplitude 2 nm and allowable external disturbance (drift rate) 0.05 nm/s. Also, indentations were applied at a target constant strain rate of 0.05 s^-1^, with a depth limit of 2 μm. For calculation, the enamel’s Poisson Ratio was set at 0.3 [38]. The ability of different remineralizing protocols to recover the elastic modulus E and nanohardness H of enamel was calculated using Equations 3 and 4 for E and H, respectively [39]:

3 $${\textrm{R}}_{\textrm{E}}\%=\left(\textrm{E}2-\textrm{E}1\right)/\left(\textrm{E}0-\textrm{E}1\right)\times 100$$

Where R_E_% : the elastic modulus recovery percentage, E0 is the elastic modulus of healthy enamel, E1 is the elastic modulus of demineralized enamel, and E2 is the elastic modulus of remineralized enamel.

4 $${\textrm{R}}_{\textrm{H}}\%=\left(\textrm{H}2-\textrm{H}1\right)/\left(\textrm{H}0-\textrm{H}1\right)\times 100$$

Where R_H_% : the nanohardness recovery percentage, H0 is the nanohardness of healthy enamel, H1 is the nanohardness of demineralized enamel, and H2 is the nanohardness of remineralized enamel.

### Statistical analysis

Data were analyzed using the statistical package for social sciences, version 23.0 (SPSS Inc., Chicago, Illinois, USA). Data were explored for normality using Kolmogorov-Smirnov and Shapiro-Wilk Tests. Two-way ANOVA was used to assess the effect of different tested variables (peptide treatment and remineralizing solutions) and their interaction. Two-way repeated-measures ANOVA was used for the analysis of surface microhardness, Raman microspectroscopy and nanoindentation test results. Bonferroni Post Hoc test was used for multiple comparisons between different variables. The statistical significance was set at α = 0.05.

## Results

### Binding capacity of FITC-labelled peptide to demineralized enamel surface

**Fig. 2:**
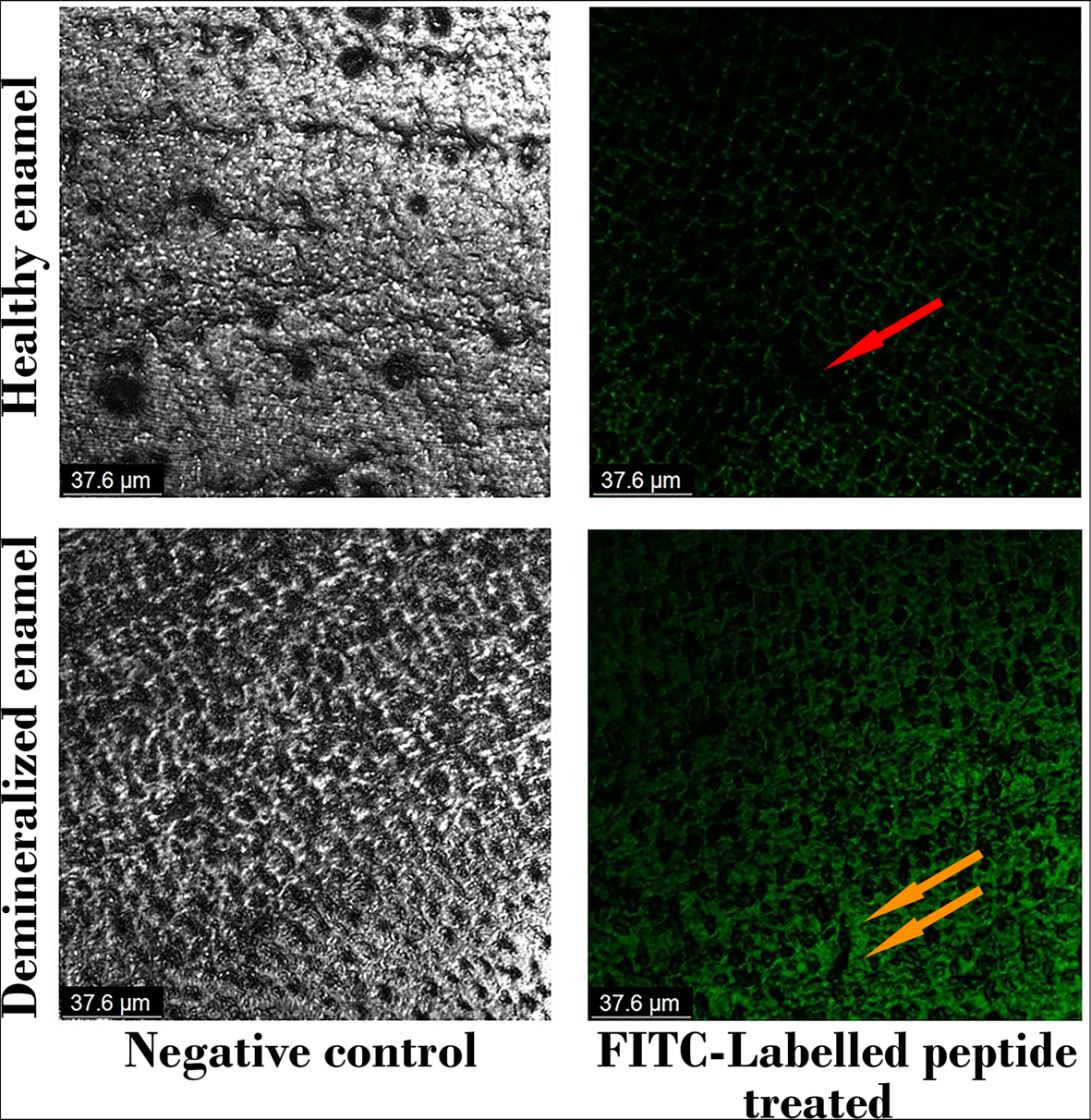
CLSM images of both control healthy and demineralized enamel as untreated (negative control) and FITC-labelled peptide-treated .Red arrow: area with low binding capacity, where peptide was washed out. Orange arrows: areas of demineralized enamel with high degree of peptide binding capacity

Figure 2 shows the fluorescent dispersion on the surface of healthy and demineralized enamel samples, before and after peptide treatment, tested by CLSM. Green fluorescence was detected, distributed sporadically on surfaces of healthy and demineralized enamel after FITC-labelled peptide treatment. Peptide could bind to 50.128 ± 4.6 % of the total area of demineralized enamel with strong fluorescence that the enamel prisms were clearly observed. On the other hand, peptide was only able to bind to 16.57 ± 0.97 % of the total area of healthy enamel. It was concluded that peptide has a good binding capacity to demineralized enamel, which would positively affect its remineralization function.

### Spontaneous mineralization testing

**Fig. 3:**
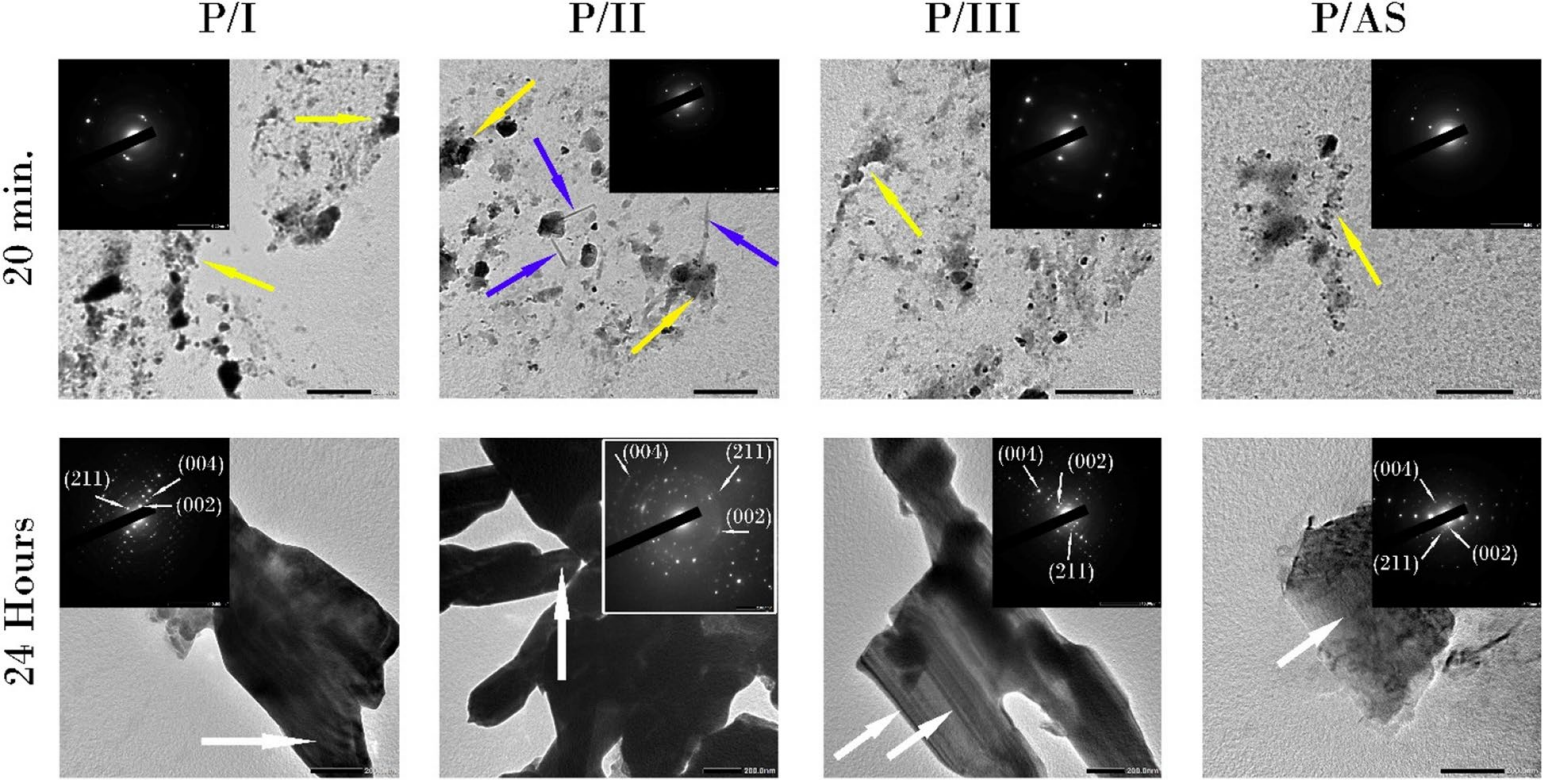
TEM images of peptide-treated groups after 20 minutes and 24 hours. Yellow arrows: agglomerated nano-spherical particles. Blue arrows: needle-like crystals in P/II. White arrows: rod-like crystals arranged in bundles. Inserts: SAED confirming the presence of a crystalline structure at 20 min and 24 h with the diffraction planes of (002), (211) and (004) labelled after 24 h

TEM images with SAED (inserts ) of peptide- treated groups are shown in Fig. 3. After 20 minutes, several agglomerates of small spherical nanoparticles were observed (yellow arrows). P/I showed denser aggregates in comparison to P/III and P/AS. In P/II, needle-like crystals started to appear after 20 minutes (blue arrows). Mineral phase identification using SAED (inserts) verified that mineral phase deposited in peptide- treated groups after 20 minutes was HAP with spotted diffraction rings.

After 24 hours, Rod-like crystals (white arrows) arranged in bundles were detected , however bundles in group P/II were denser, that few rods can be clearly seen. SAED (inserts) clearly showed the HAP with stronger diffraction rings and labelled with diffraction planes of (002), (211) and (004).

**Fig. 4:**
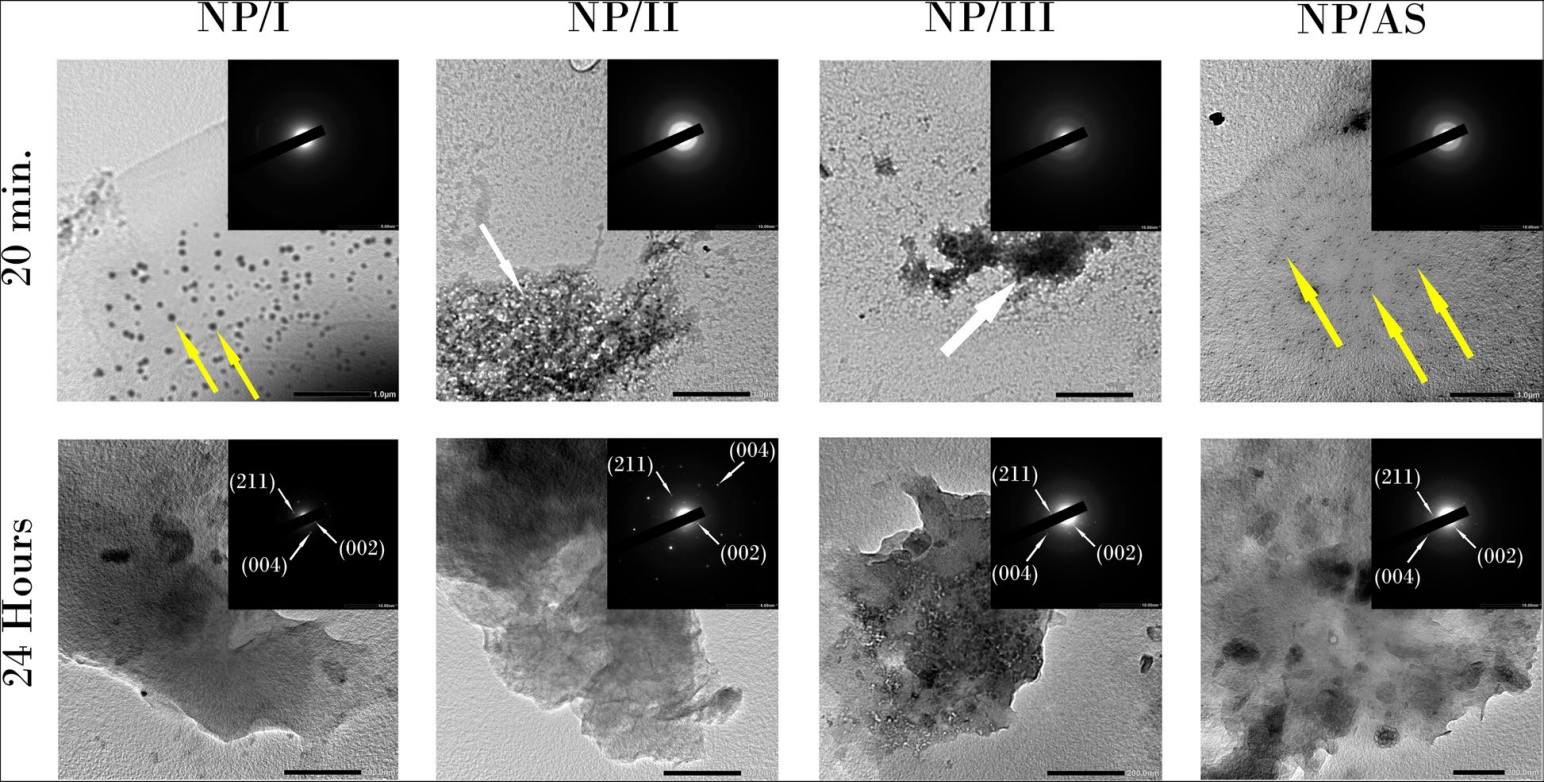
TEM images of non-peptide treated groups after 20 minutes and 24 hours. Yellow arrows: randomly distributed spherical ACP particles in NP/I and NP/AS. White arrows: aggregated ACP nanoparticles in NP/II and NP/III. Inserts: SAED confirming the presence of a crystalline structure with the diffraction planes of (002), (211) and (004) labelled after 24 hours

TEM image of non-peptide treated groups are shown in Fig. 4. Small spherical ACP (amorphous calcium phosphate) particles were seen in all non-peptide treated groups as verified with SAED (inserts) where diffuse diffraction rings were detected. In NP/I and NP/AS, ACP particles were randomly distributed (yellow arrows), while in NP/II and NP/III, nanoparticles appeared grouped together (white arrows). After 24 hours, film-like HAP crystals were detected in all groups as confirmed by spot diffraction rings in SAED.

### Structural characterization

#### XRD analysis and preferential crystal orientation

**Fig. 5:**
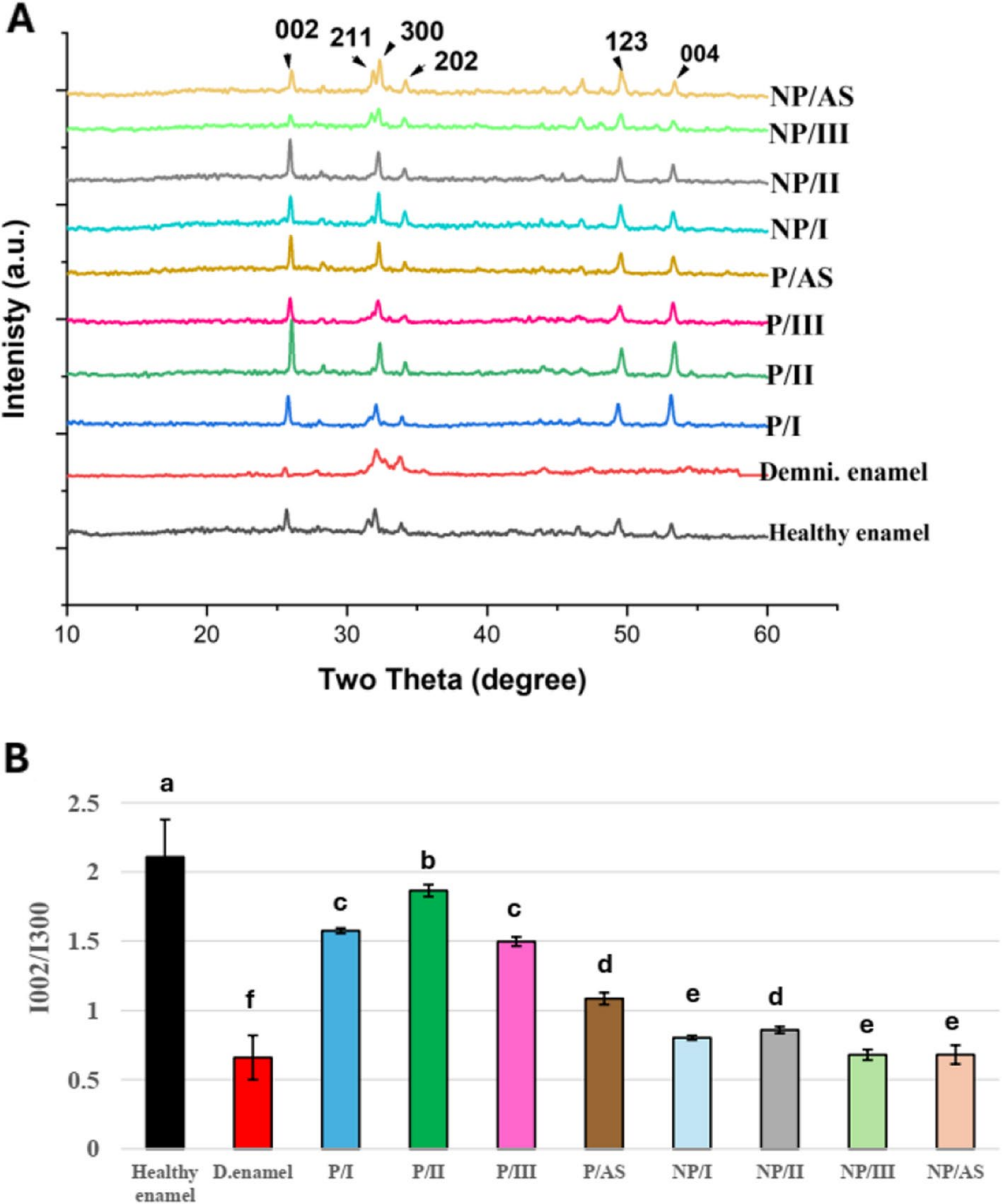
**A** representative XRD spectra of enamel surfaces of control healthy, demineralized enamel and remineralized enamel in different experimental groups after pH-cycling regimen, labelled with major peaks of HAP. **B** Bar chart of I_002_:I_300_ of remineralized enamel in different experimental groups in comparison to healthy and demineralized control groups, different letters indicate significant difference at *P*<0.05

Figure 5A depicts XRD diffraction peaks of control healthy, demineralized enamel and remineralized enamel in different experimental groups. All groups showed peaks that match the ICDD no.09-0432 for HAP at 2θ =25.9° (002), 31.86° (211), 32.9° (300), 34.023° ( 202), 49.51° (123) and 55.205° (004) [21, 26]. Figure 5B shows mean I_002_:I_300_ for control healthy, demineralized enamel and 8 remineralized experimental groups, representing the degree of orientation along the c-axis. A significant decrease  (*P* <0.001) in I_002_:I_300_ was detected in the control demineralized enamel group (0.37**±**0.07) in comparison to the control healthy enamel (2.56±0.59). All remineralizing groups were able to significantly enhance I_002_:I_300_ in comparison to the demineralized enamel (*P* <0.05), despite being significantly lower than control healthy enamel (*P* <0.001). Two- way ANOVA and Bonferroni-corrected post hoc test for pairwise comparisons revealed that all peptide-treated groups (P/I, P/II, P/III and P/AS) had significantly higher I_002_:I_300_ (1.61±0.06, 1.87±0.08, 1.42±0.14 and 1.12±0.06), respectively, than their corresponding alternatives in non-peptide treated groups (NP/I, NP/II, NP/III and NP/AS) with their I_002_:I_300_ values (0.73±0.05, 0.94±0.05, 0.73 ±0.03 and 0.66 ±0.02) respectively.

#### Raman spectral analysis and mineral content

Raman intensities of (PO_4_^3-^) peak in different remineralizing groups for healthy (I-baseline), demineralized (I-lesion) and remineralized enamel (I-post) are shown in Table 2. A statistically significant decrease was detected in Raman intensities of (PO_4_^3-^) peak in all groups after demineralization (I-lesion). All remineralizing protocols in different experimental groups were able to significantly increase Raman intensities of (PO_4_^3-^) peak (I-post) when compared to recorded (I-lesion). However (I-post) in all groups was still significantly lower than their (I-baseline). All peptide treated groups showed significantly higher Raman intensity of (PO_4_^3-^) peak in comparison to their alternatives in non-peptide treated groups.

**Table 2:** Mean ± Standard deviation values of Raman intensity of (PO_4_^3-^) peak in different remineralizing groups

Groups		I-baseline	I-lesion	I-post
**Peptide**	**P/I**	1039.78 ^Aa^ ±67.45	205.57 ^Ca^ ±13.86	803.79 ^Bb^ ±63.11
	**P/II**	1101.08 ^Aa^ ±77.12	200.45 ^Ca^ ±6.34	894.50 ^Ba^ ±18.89
	**P/III**	1014.96 ^Aa^ ±55.10	201.36 ^Ca^ ±9.82	707.17 ^Bc^ ±24.69
	**P/AS**	1007.68 ^Aa^ ±59.03	197.35 ^Ca^ ± 9.46	491.38 ^Bd^ ±26.37
**Non-Peptide**	**NP/I**	1032.34 ^Aa^ ±49.99	205.93^Ca^ ± 11.11	473.01 ^Bd^ ±16.34
	**NP/II**	1012.79 ^Aa^ ±35.31	200.96 ^Ca^ ±9.43	695.33 ^Bc^ ±30.61
	**NP/III**	1051.22 ^Aa^ ±59.12	202.57 ^Ca^ ±11.55	409.92 ^Be^ ±12.69
	**NP/AS**	1025.61 ^Aa^ ±61.41	200.05 ^Ca^ ±10.00	218.29 ^Bf^ ±10.65

Results of two-way repeated measures ANOVA and Bonferroni-corrected post hoc test for pairwise multiple comparisons. Different uppercase and lowercase superscript letters indicate significant differences among the same raw and column, respectively at *P* <0.05

**Fig. 6:**
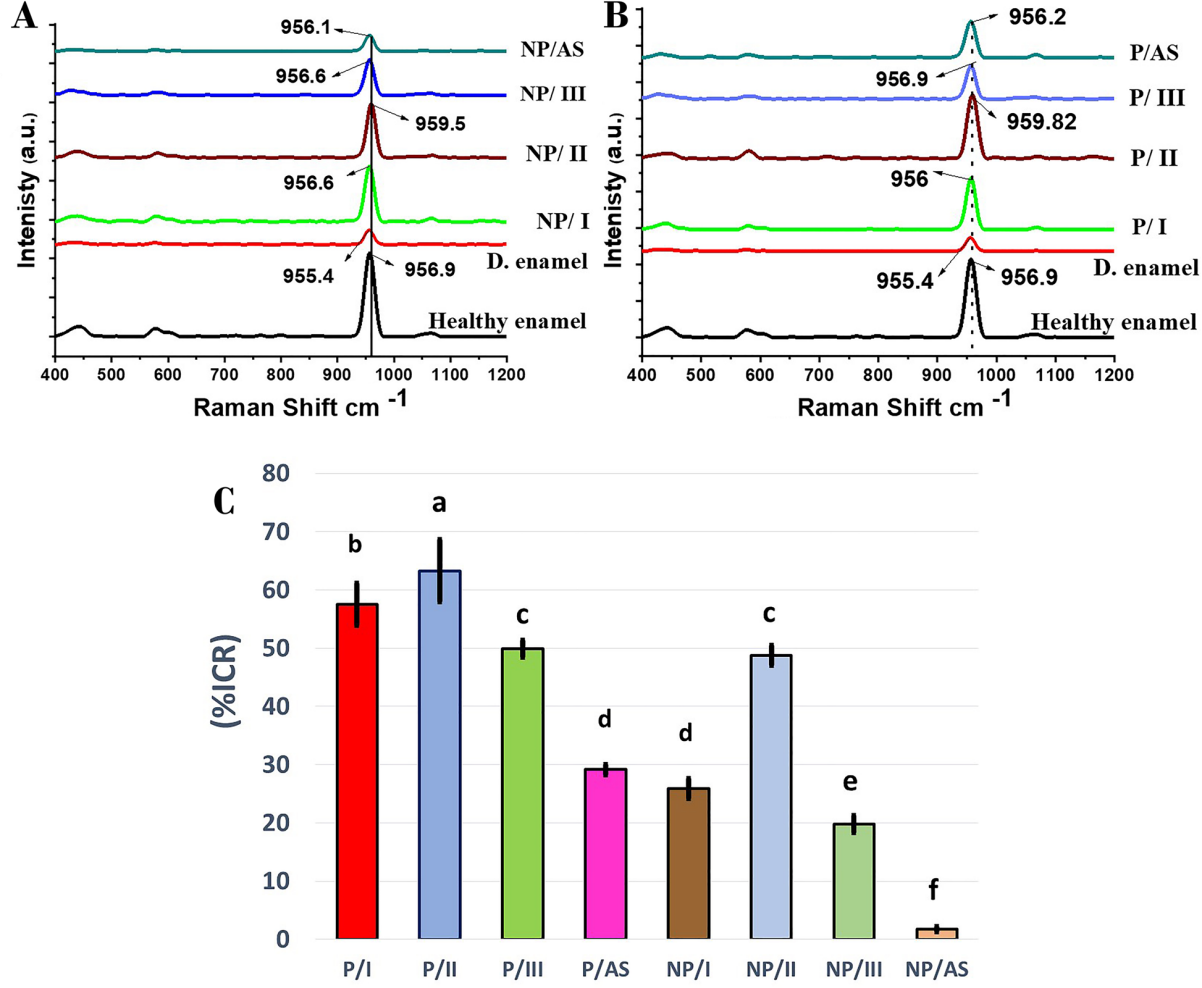
**A** representative Raman spectra of non-peptide treated groups in comparison to control healthy and demineralized enamel showing the exact position of (PO_4_^3-^) peak, solid line; indicates a ~3 cm^-1^ shift to higher value in NP/II. **B** representative Raman spectra of peptide-treated groups compared to control healthy and demineralized enamel showing the exact position of (PO_4_^3-^) peak, dotted line indicates a ~3 cm^-1^ shift to higher value in P/II. **C** Mean Raman intensity change ratio (%ICR) of the different remineralizing groups. Different letters demonstrate statistically significant difference (*P*< 0.05)

Figure 6 (A and B) shows the most intensive ν1 (PO_4_^3-^) peak that arises from the symmetric P–O bond stretches in the surface mineral layer of enamel in different remineralizing groups. The exact value of (PO_4_^3-^) peak position was specified. A clear upshift (to the right) of the band position was detected in groups NP/II (Fig. 6A, solid vertical line) and P/II (Fig. 6B, dotted vertical line) to higher values (959.5 and 959.82 cm^-1^), respectively.

Raman intensity change ratios (%ICR) for the different groups are shown in Fig. 6C. A statistically significant difference was detected between groups (*P* <0.001) regarding (%ICR) using Two- way ANOVA and Bonferroni-corrected post hoc test for pairwise comparisons. P/II showed the highest statistically significant recovery in surface mineral content (63.31% ±5.32) among all experimental groups, followed by P/I (57.51% ± 3.59). NP/II showed the statistically significant highest recovery in surface mineral content among non-peptide treated groups (48.80%± 1.73) with no statistical difference with P/III (49.88% ±1.46). P/AS and NP/I showed no statistically significant difference in their calculated mineral content recovery percentage (29.15% ±0.91, 25.92 %±1.68), respectively. NP/III recorded a significantly lower ICR% (19.79% ±1.41). The statistically significant least ICR% was calculated in NP/AS (1.78% ±0.47).

### Surface, cross-sectional morphology and elemental composition

**Fig. 7:**
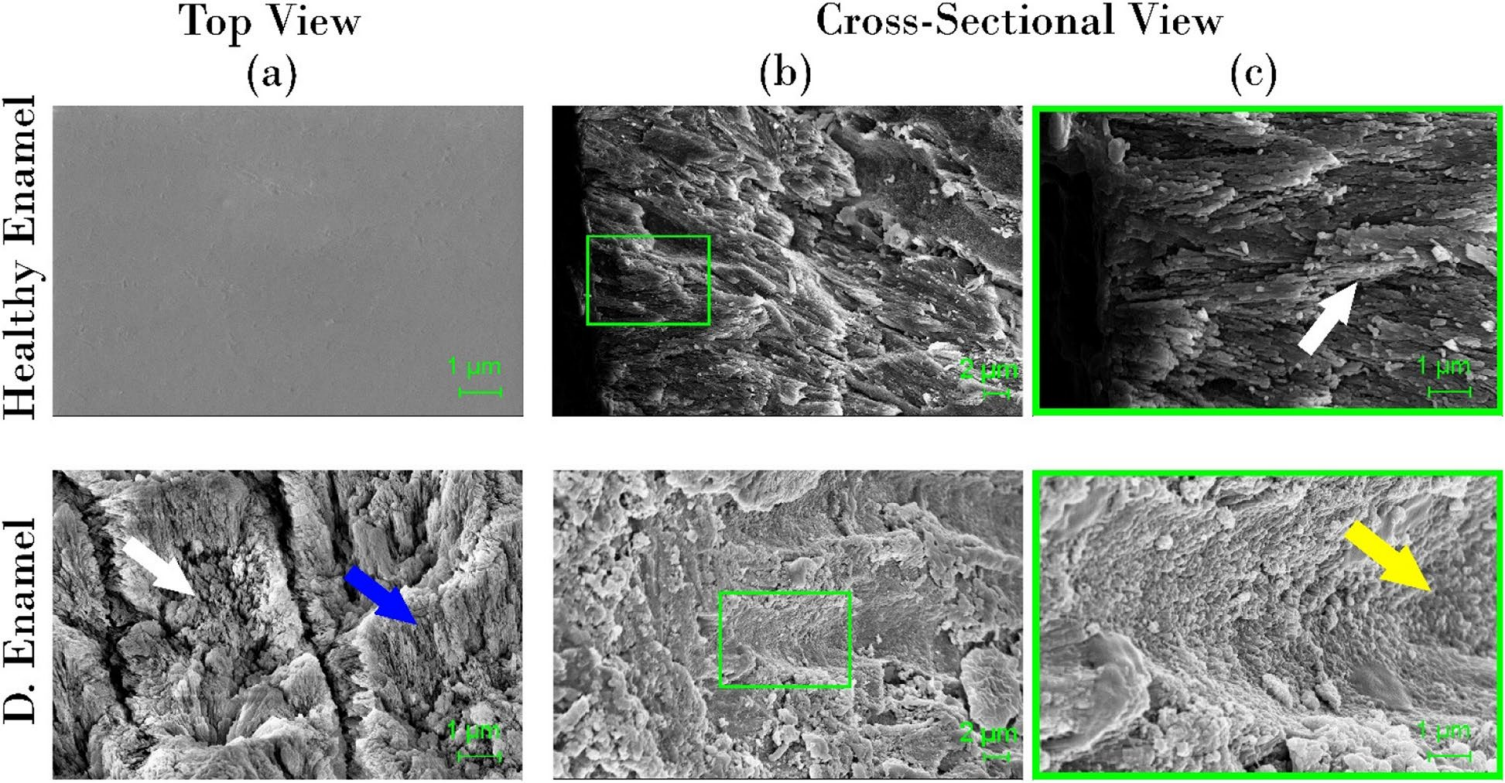
SEM micrographs of control healthy and demineralized enamel (top- view (**a**) and cross-sectional view (**b**) at 3000X and (**c**) Magnified image of the square in panel of (**b**) at 10000 X. white arrow in healthy enamel (**c**): intact enamel rod. White arrow: in demineralized enamel (**a**): deformed rods and crystallites exposed on the surface of demineralized enamel, blue arrow: interrod sheath. yellow arrow: deformed eroded rod

The micrographs of control healthy and demineralized enamel surfaces are shown in Fig. 7. Healthy enamel showed normal intact enamel surface and well-aligned enamel rods (white arrow) with HAP crystallites displayed in the cross-sectional view. Micrographs of demineralized enamel surface show deformed enamel rods (white arrow) interrod sheath (blue arrow). In the cross-sectional view, deep eroded areas, marked with yellow arrow,  were detected.

**Fig. 8:**
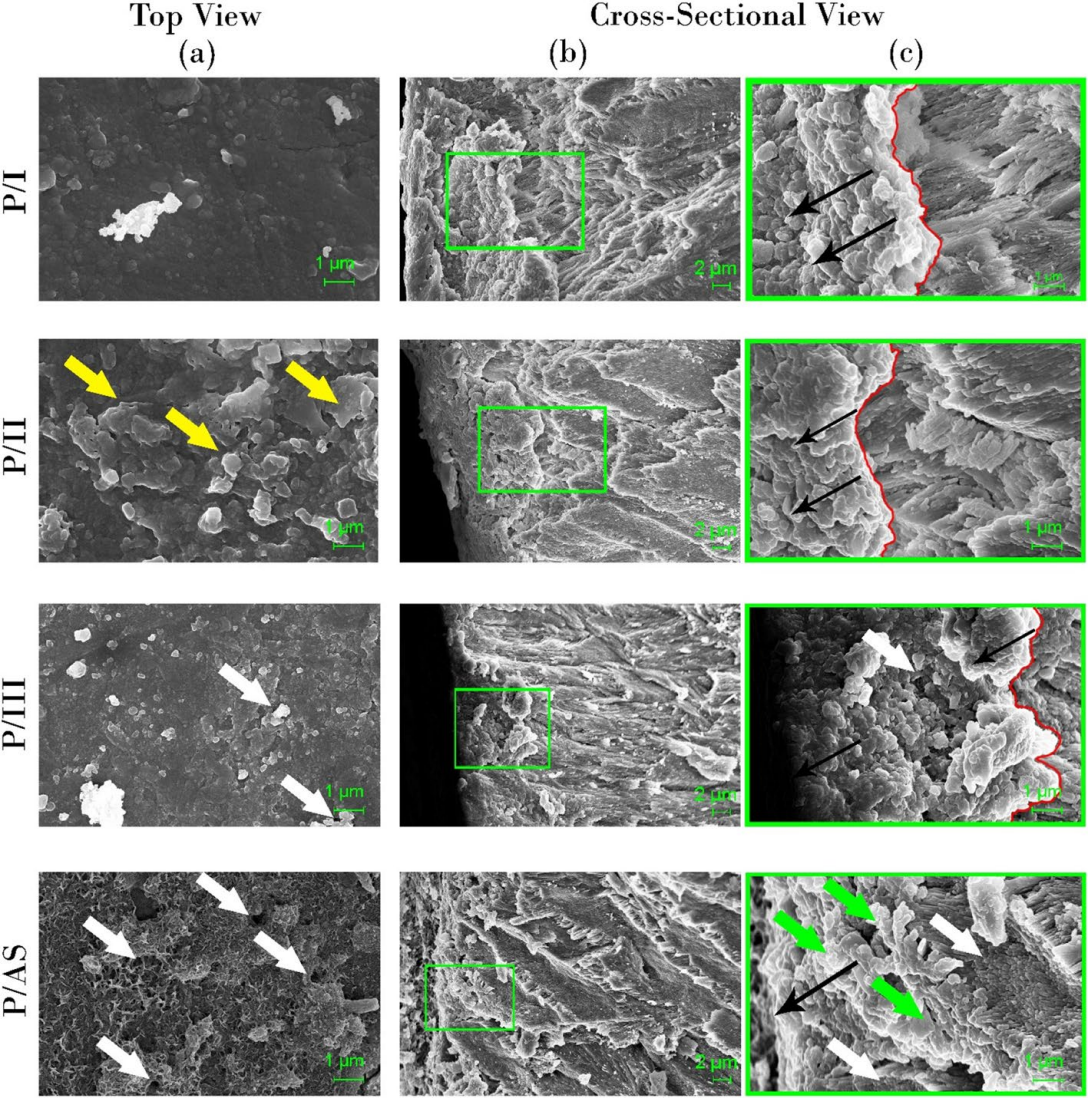
SEM micrographs of peptide-treated groups (top- view (**a**) and cross-sectional view (**b**) at 3000X and (**c**) Magnified image of the square in panel of (**b**) at 10000 X. Red line; interface between native enamel rods and the newly formed apatite layer in P/I, P/II and P/III. Yellow arrows; thicker areas in mineral layer in P/II. Black arrows; designate the direction of growth of mineral crystals. White arrows; discontinuations and clefts exposing enamel underneath in P/III and P/AS. Green arrow; discrete deposits in P/AS

Figure 8 shows SEM micrographs of peptide-treated groups (P/I, P/II, P/III, P/AS). P/I and P/II formed a continuous mineral layer on demineralized enamel, with thicker patches (yellow arrows) in P/II (top view). In P/III, discontinuations (white arrow) were detected in the mineral layer. Also, in P/AS, many discontinuations (white arrows) were detected all over the surface with discrete mineral deposits. In the cross-sectional view, small crystallites appear clumped together in a directional manner, marked by black arrows in all 4 groups. The red lines mark the interface between native enamel rods and the newly formed mineral layer in P/I, P/II and P/III, while discrete mineral deposited on the surface in P/AS are marked with green arrows. The white arrows in P/III and P/AS mark the exposed underlying enamel rods not covered with the new mineral deposits.

**Fig. 9:**
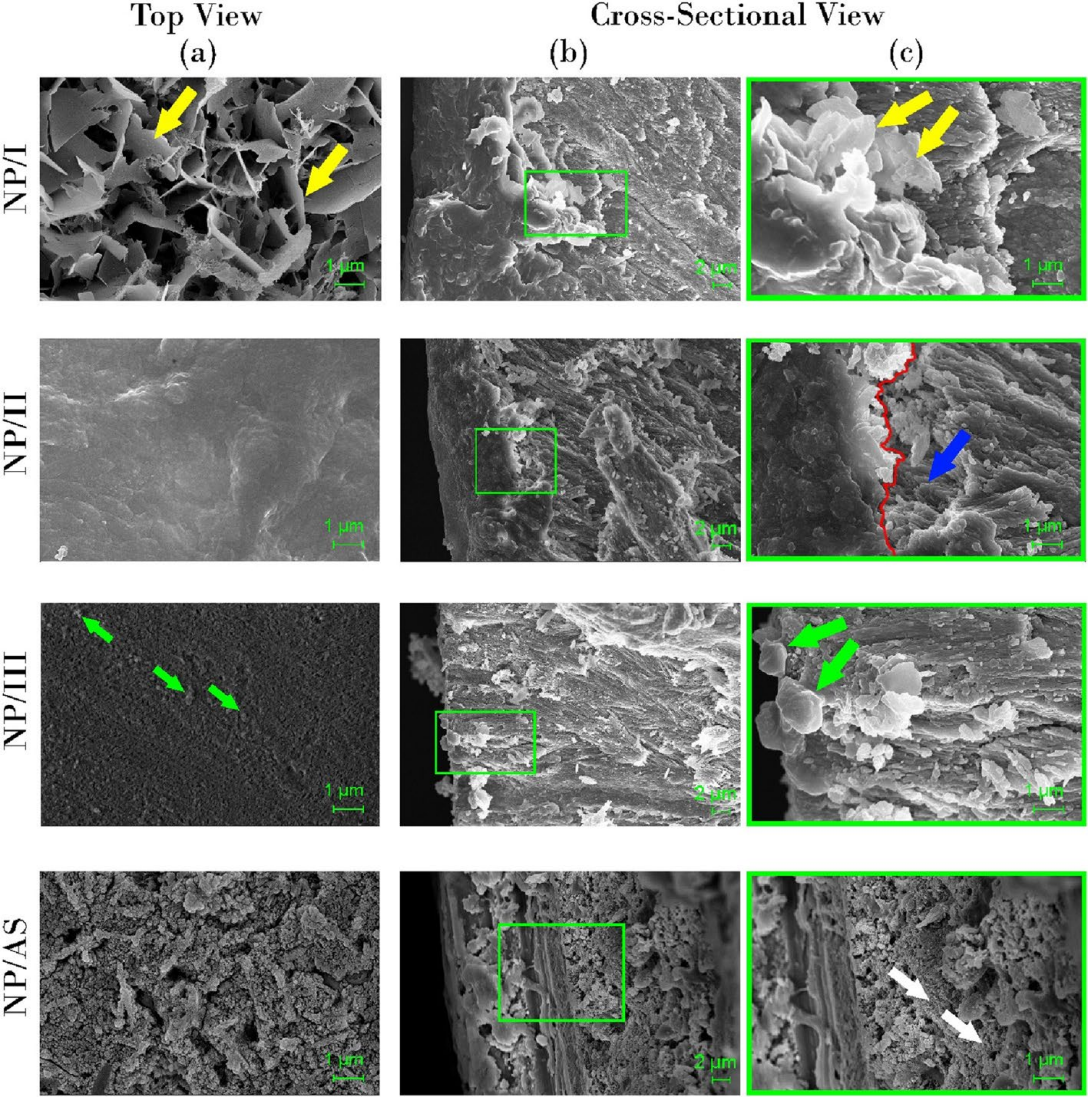
SEM micrographs of non- peptide treated groups (top- view (**a**) and cross-sectional view (**b**) at 3000X and (**c**) Magnified image of the square in panel of (**b**) at 10000 X . Yellow arrows; thin plates detected covering the demineralized enamel surface in top view with its remnants in cross-sectional view of NP/I. NP/II; red line; interface between dense mineral layer and the underlying enamel, blue arrow; area of separation between the mineral layer and native enamel. Green arrows; discrete deposits in NP/III. White arrows; eroded deformed enamel rods in NP/AS

SEM micrographs of non-peptide-treated groups are shown in Fig. 9. In group NP/I, the top- view focused on areas with thin plates (yellow arrows) covering the demineralized enamel surface. In the cross-sectional view, remnants of these plates were observed below the formed mineral layer (yellow arrows). SEM micrographs of group NP/II revealed a dense mineral layer masking the native demineralized enamel. However, in the cross-sectional view, the mineral layer (marked by a red line) appeared separated from the underlying enamel prism with pores in specific areas (blue arrow). In group NP/III, only few mineral deposits were detected in both the top view and cross-sectional view (green arrows), exposing the demineralized enamel underneath. Both top and cross-sectional views in NP/AS showed exposed deformed demineralized enamel (white arrows).

**Fig. 10:**
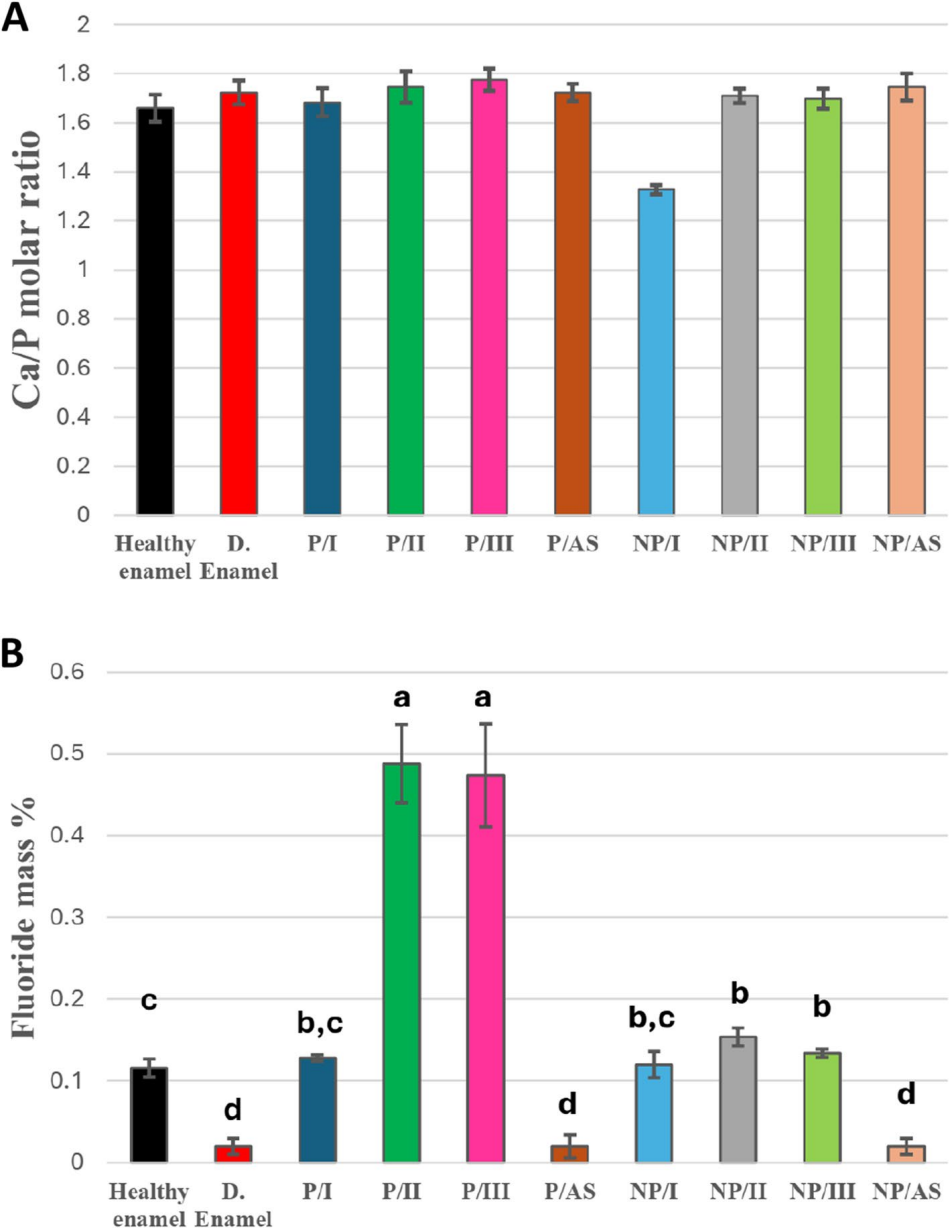
**A** Bar charts showing mean Ca/P molar ratio of enamel indifferent remineralizing groups. **B **Bar charts showing mean fluoride mass % of control healthy, demineralized enamel and different remineralizing groups, groups with different lowercase letters are significantly different at *P*<0.05

Ca/P molar ratio of control healthy and demineralized enamel and remineralized enamel in different groups (Fig. 10A) were calculated from Ca and P mass % in EDXS results. All remineralized enamel samples in different groups showed a Ca/P molar ratio near to that of healthy enamel (1.666±0.055) except NP/I. Peptide treated groups (P/I, P/II, P/III and P/AS) had Ca/P molar ratios of (1.684±0.057, 1.746±0.064, 1.776±0.045 and 1.724±0.035) respectively. For non-peptide treated groups, Ca/P molar ratios were 1.328±0.019 for NP/I, 1.718±0.029 for NP/II, 1.698±0.041 for NP/III and finally 1.746±0.055 for NP/AS. Figure 10B displays the fluoride mass % of different remineralizing groups compared to control healthy and demineralized enamel, where a significant difference was reported (*P*<0.001). Different remineralizing groups showed a significant increase in fluoride mass % compared to the control demineralized group (0.020±0.01), except P/AS and NP/AS. P/II and P/III had the highest significant values (0.488±0.048 and 0.474±0.063 respectively), followed by NP/II (0.154±0.011) and NP/III (0.134±0.005) and finally, P/I and NP/I with fluoride mass % of 0.128±0.004 and 0.120±0.016, respectively.

### Mechanical properties measurement

#### Surface microhardness and surface microhardness recovery ratio

**Fig. 11:**
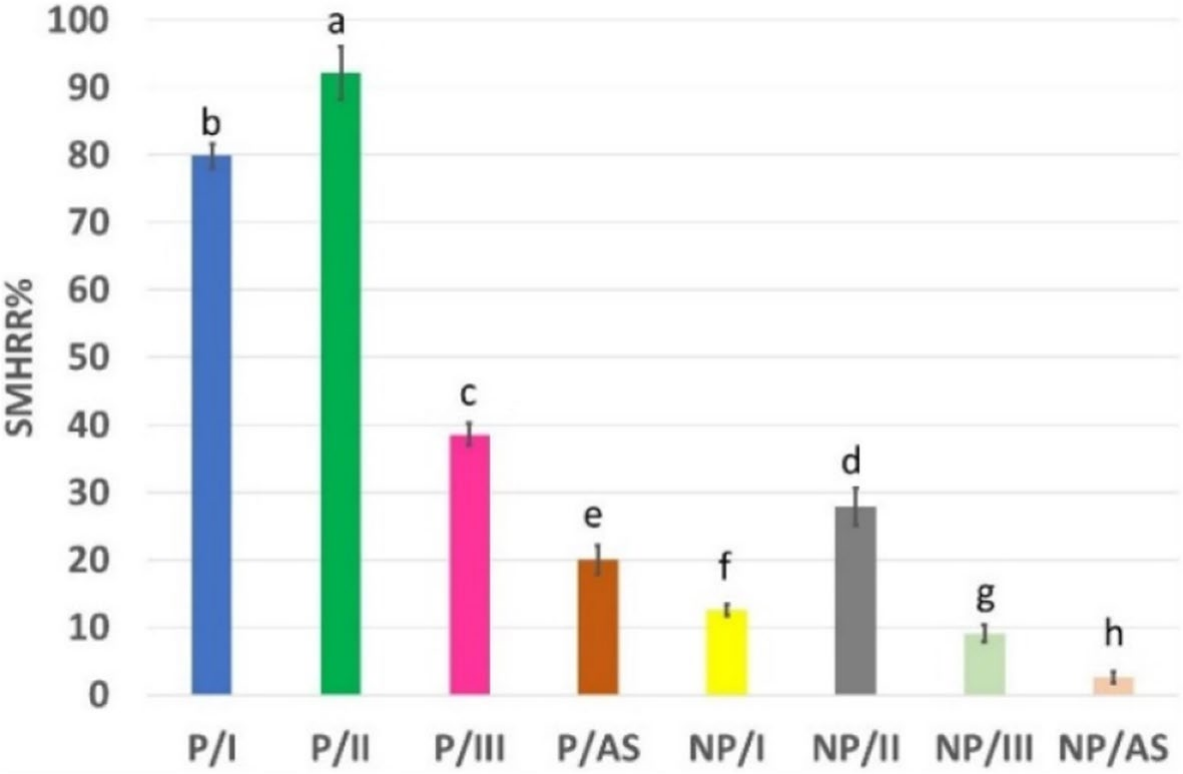
Bar chart showing mean surface microhardness recovery ratio SMHRR% of different remineralizing groups. Different lowercase letters demonstrate statistically significant difference (*P* <0.05)

Table 3 shows the SMH of healthy (SMH0), demineralized (SMH1) and remineralized enamel (SMH2) in different groups. No significant difference was detected between all groups regarding SMH0 and SMH1 of enamel. For each of the tested experimental groups, SMH0 of healthy enamel revealed the highest significant mean value, followed by SMH2 after pH-cycling, and the lowest significant value was recorded after demineralization SMH1 (*P*<0.05). A statistically significant difference in SMH2 (*P*<0.001) was detected between different groups. Bonferroni-corrected post hoc test for pairwise comparisons showed a statistically significant difference in SMHRR% between the experimental groups (*p*<0.001), as shown in Fig. 11. P/II showed the statistically significant highest SMHRR% and was able to recover enamel surface microhardness by 92.19% ± 3.91 of that of healthy enamel , followed by P/I with 79.90 % ± 1.76 recovery percentage.

**Table 3:** Mean ± standard deviation values of surface microhardness (VHN) in different remineralizing groups

Groups		SMH0	SMH1	SMH2
**Peptide**	**P/I**	281.29 ^Aa^ ±5.33	76.43 ^Cb^ ±4.41	240.04 ^Bb^ ±2.43
	**P/II**	285.23 ^Aa^ ±8.13	76.59 ^Cb^ ±3.19	268.81 ^Ba^ ±6.52
	**P/III**	268.88 ^Aa^ ±14.07	76.48 ^Cb^ ±3.22	150.89 ^Bc^ ±5.18
	**P/AS**	277.54 ^Aa^ ±15.22	76.67 ^Cb^ ±3.97	116.81 ^Be^ ±5.38
**Non-Peptide**	**NP/I**	282.98 ^Aa^ ±10.54	77.28 ^Cb^ ±2.68	103.09 ^Bf^ ±3.61
	**NP/II**	275.78 ^Aa^ ±15.28	77.01 ^Cb^ ±3.37	132.54 ^Bd^ ±6.24
	**NP/III**	271.78 ^Aa^ ±12.06	75.10 ^Cb^ ±2.78	92.85 ^Bg^ ±2.73
	**NP/AS**	278.39 ^Aa^ ±15.62	76.46 ^Cb^ ±3.30	81.80 ^Bh^ ±3.00

Different uppercase and lowercase superscript letters indicate significant difference (*P*<0.05) among the same raw and column, respectively

#### Nanomechanical properties

Tables 4 and 5 show the mean elastic modulus E and nanohardness H of healthy, demineralized and remineralized enamel in different groups. No statistical difference was detected between different groups regarding elastic modulus and nanohardness of healthy (E0, H0) and demineralized enamel (E1, H1). All remineralizing protocols were able to significantly enhance both E2 and H2 of enamel in comparison to demineralized enamel, despite being statistically lower than healthy enamel. A highly significant difference was detected in E2 and H2 between different groups (*p*<0.05).

**Table 4:** Mean ± standard deviation values of elastic modulus (GPa) in different remineralizing groups

Groups		E0	E1	E2
**Peptide**	**P/I**	134.86 ^Aa^ ±17.87	4.38 ^Ca^ ±2.14	73.14 ^Bb^ ±4.41
	**P/II**	119.04 ^Aa^ ±14.66	3.68 ^Ca^ ±2.27	88.74 ^Ba^ ±2.71
	**P/III**	116.48 ^Aa^ ±12.03	4.84 ^Ca^ ±3.15	66.96 ^Bc^ ±2.09
	**P/AS**	122.80 ^Aa^ ±18.70	4.78 ^Ca^ ±1.93	55.06 ^Bd^ ±3.24
**Non-Peptide**	**NP/I**	120.80 ^Aa^ ±17.11	3.98 ^Ca^ ±1.56	31.62 ^Bf^ ±4.58
	**NP/II**	117.56 ^Aa^ ±11.81	4.60 ^Ca^ ±1.76	41.90 ^Be^ ±1.93
	**NP/III**	120.35 ^Aa^ ±13.35	4.66 ^Ca^ ±2.34	18.24 ^Bg^ ±1.27
	**NP/AS**	123.56 ^Aa^ ±15.60	4.64 ^Ca^ ±1.82	14.88 ^Bg^ ±1.67

Different uppercase and lowercase superscript letters indicate significant difference among same row and column, respectively at *P*<0.05

**Table 5:** Mean ± standard deviation values of nanohardness (GPa) in different remineralizing groups

Groups		H 0	H 1	H 2
**Peptide**	**P/I**	4.44 ^Aa^ ±1.18	0.07 ^Ca^ ±0.04	2.69 ^Bb^ ±0.12
	**P/II**	3.52 ^Aa^ ±0.58	0.07 ^Ca^ ±0.04	3.08 ^Ba^ ±0.59
	**P/III**	3.06 ^Aa^ ±0.79	0.11 ^Ca^ ±0.07	1.80 ^Bc^ ±0.20
	**P/AS**	3.34 ^Aa^ ±0.57	0.09 ^Ca^ ±0.03	1.25 ^Bd^ ±0.23
**Non-Peptide**	**NP/I**	3.72 ^Aa^ ±0.79	0.07 ^Ca^ ±0.05	0.63 ^Be^ ±0.05
	**NP/II**	3.26 ^Aa^ ±0.59	0.05 ^Ca^ ±0.03	1.15 ^Bd^ ±0.13
	**NP/III**	3.10 ^Aa^ ±0.48	0.10 ^Ca^ ±0.06	0.30 ^Bf^ ±0.06
	**NP/AS**	3.08 ^Aa^ ±0.54	0.08 ^Ca^ ±0.02	0.27 ^Bf^ ±0.06

Different uppercase and lowercase superscript letters indicate significant difference among same row and column, respectively at *P*<0.05

**Fig. 12:**
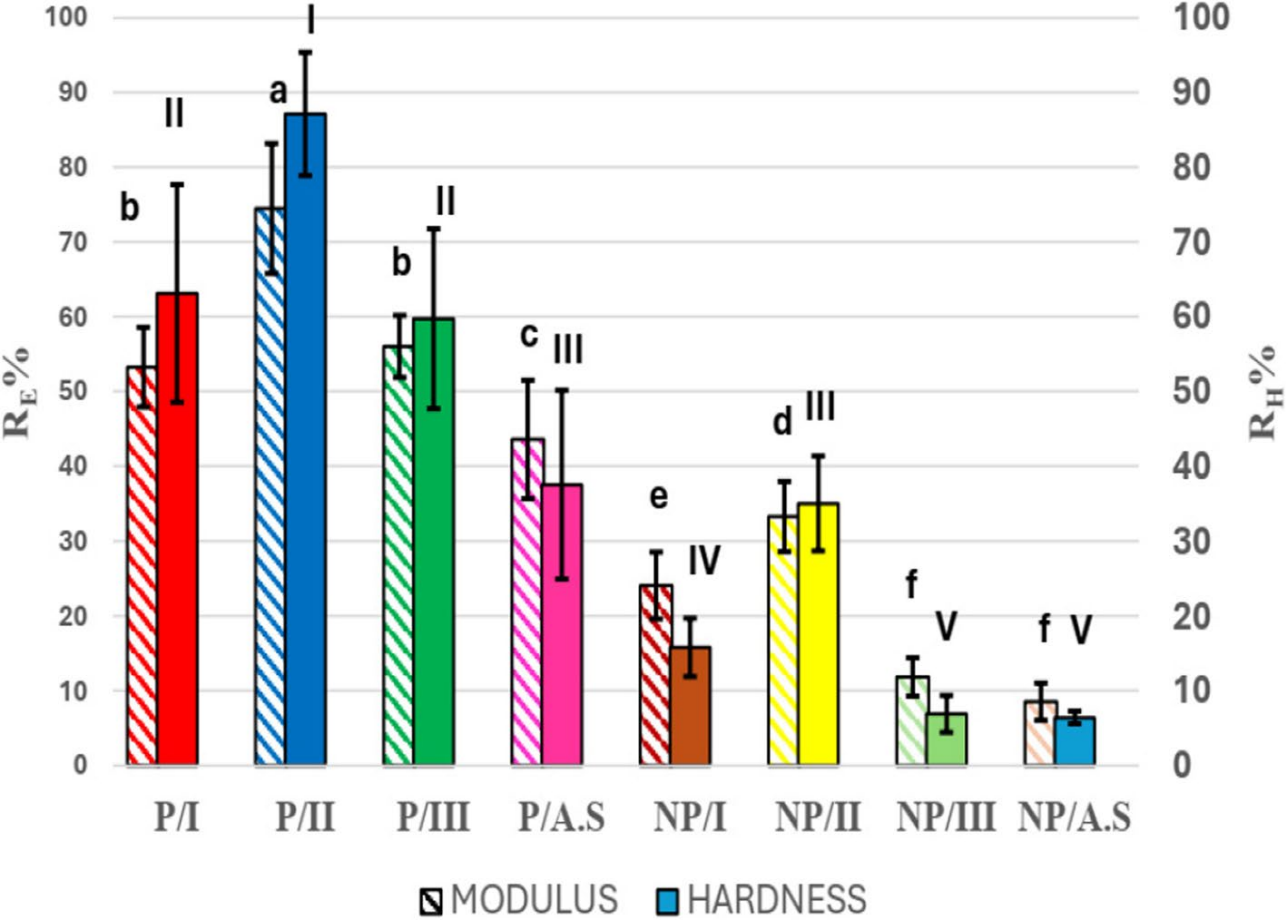
Bar chart showing mean elastic modulus Recovery percentage (R_E_ %) and mean nanohardness recovery percentage (R_H_ %) of different remineralizing groups. Different letters demonstrate statistically significant difference in R_E_ % (*P* < 0.05). Different roman numbers demonstrate statistically significant difference in R_H_ % (*P* < 0.05)

Figure 12 shows the recovery percentage of both elastic modulus (R_E_ %) and nanohardness (R_H_ %) in different groups. All peptide-treated groups were significantly higher than their corresponding alternatives in non-peptide-treated groups regarding R_E_% and R_H_%. Group P/II showed the highest statistically significant recovery % in both E (R_E_% 74.51±8.70) and H (R_H_% 87.16±8.25) , followed by both P/I (R_E_% 53.27±5.33 and R_H_% 63.14±14.56) and P/III (R_E_% 56.05±4.15 and R_H_% 59.75±12.03). P/AS showed the significantly least values in peptide treated groups with R_E_% equals 43.62±7.90 and R_H_% equals 37.58±12.61. NP/II had the highest significant mean value among non-peptide groups (R_E_% 33.33±4.70 and R_H_% 35.06±6.32) followed by NP/I (R_E_% 24.11±4.47 and R_H_% 15.83±3.91), then NP/III (R_E_% 11.87±2.57 and R_H_% 6.92±2.47) and NP/AS (R_E_% 8.56±2.49 and R_H_% 6.45±0.84) with no significant difference between both groups.

## Discussion

Dental enamel is the hardest mineralized tissue in the human body with its unique strength, anti-erosive and abrasive properties. It has more than 95 % mineral content in the form of nanorod-like hydroxyapatite crystals that are arranged in a highly organized hierarchical microstructure (prism). This unique hierarchical arrangement was mediated by proteins, such as amelogenin and ameloblastin, secreted by ameloblasts during enamel biomineralization. Therefore, one of the attempts of biomimetic remineralization was to develop amelogenin analogues that mimic the functional domain of amelogenin [5].

In the current study, this peptide was designed by Mukherjee et al. mimicking and translating the amelogenin function in biomineralization. This amino acid sequence provided a hydrophilic negatively charged peptide that was able to assemble into nanospheres when observed with transmission electron microscope, as reported by Mukherjee et al. [21]. The last 12 amino acid of this peptide were preserved from the hydrophilic C-terminus of the amelogenin. The hydrophilic C-terminal plays a control role in peptide–apatite interaction [40, 41]. This is attributed to the charged side chains of Lysine (K) and Aspartic acid (D) that allow direct ionic interaction with calcium and phosphate and subsequently initializing hydroxyapatite nucleation with oriented growth of enamel crystals [40, 41]. Additionally, this peptide was designed to preserve 14 amino acid residues form the inner N-terminus of amelogenin (residues 1-4; 16-25). The N-terminus plays a major role in peptide self-assembly and mineralization kinetics [21].

A supersaturated calcium phosphate remineralizing solution was used in the current study to be tested alone and in conjugation with the peptide. It was produced with an initial molar ratio of Ca^2+^ to PO_4_^3-^ ions equals 1.67 with a relatively high supersaturation degree that would thermodynamically accelerate the nucleation of crystals by precipitating numerous Ca-P clusters [11, 42]. It was interesting to investigate the ability of fluoride to enhance and augment the remineralizing potential of the peptide and the remineralizing solution and to evaluate the mineral layer developed using such a combination. A fluoride concentration of 1100 ppm was used, representing the most common concentration in commercially available toothpaste [19].

TEM images of peptide-treated groups (Fig. 3), in comparison to non-peptide treated groups (Fig. 4), confirmed the ability of the tested peptide to stabilize the ACP precursors to crystallize into HAP within 20 minutes. Simple spot diffraction pattern was detected in SAED of peptide-treated groups, unlike non-peptide treated groups where diffuse diffraction pattern was observed. Furthermore, after 24 hours, TEM images of peptide-treated groups highlighted that the peptide acted as a template to induce the formation of rod-like HAP crystals in a way resembling the ribbon-like apatite minerals observed in developing enamel [43, 44].

SEM images of non-peptide treated groups are shown in Fig. 9. Group NP/I showed a layer of thin plates deposited on the enamel surface. This observation was in agreement with Teerakanok et al. [11] and Sugaya et al. [45] in their research, where they reported the deposition of thin plates of octa calcium phosphate (OCP), a metastable precursor of HAP, on remineralized enamel surfaces. OCP has the ability to transform to stable HAP, guided by the structural similarity between both phases, as OCP has the same unit cell of HAP [46, 47]. This observation was further supported by EDX and XRD. EDX analysis (Fig. 10A) recorded a Ca/P molar ratio of 1.328, in agreement with Teerakanok et al. [11], who reported that the theoretical Ca/P molar ratio of OCP equals = 4/3 (1.33) [11, 48]. OCP and HAP share the major characteristic peaks in XRD (002), (211) and (300) (Fig. 5A) [46, 47]. According to literature, XRD pattern with reflections at 4.74, 9.44 and 9.76 2θ corresponding to crystal planes (100), (200) and (010), respectively, are characteristic for OCP [48]. However, the XRD pattern of NP/I did not show these peaks, which was also in parallel with Teerakanok et al. [11]. According to Kajiyama et al. [49], the absence and disappearance of 010 reflection of OCP might be related to the size of the nanocrystallites.

No evidence of OCP was detected with the addition of 1100 ppm of fluoride to the calcium phosphate solution in group NP/II. However, a dense layer of minerals deposit was found to cover the surface of demineralized enamel (Fig. 9). This newly formed mineral layer is supposed to be HAP with a Ca/P molar ratio of 1.718 (Fig. 10A) and major XRD peaks of HAP (Fig. 5A) [11, 19, 21]. Lijima et al .[47] reported that fluoride decreases the cluster stability needed for OCP growth from calcium phosphate clusters while increasing the stability needed for HAP growth. Other researchers used fluoride as a promoter for the conversion of metastable OCP into HAP and fluorapatite crystals [11, 46, 50]. This might explain the presence of HAP in NP/II and OCP in NP/I. The addition of fluoride resulted in a significant increase in I_002_:I_300_ ratio from 0.73 in NP/I to 0.94 in NP/II (Fig. 5B), translated as an increase in 002 diffraction peak [21]. This indicates that fluoride accelerated the growth of HAP in group NP/II in preferential orientation along the c-axis, perpendicular to 002 planes.

Fluo-hydroxyapatite (F-HAP) and fluorapatite (FAP) are obtained when OH^-^ ions in HAP are partially or completely substituted by F^-^ ions [51, 52]. The Raman spectral data of NP/II showed a shift of ~3cm^-1^ in the (PO_4_^3-^) peak position in comparison to healthy enamel (Fig. 6A). According to Seredin et al. [52], OH^-^ substitution with F^-^ in HAP supported by proximity of the radii of ions, resulting in a shift of the Raman scattering band ν1 (PO_4_^3-^) to higher frequencies. Also, Yu et al .[53] in their study investigating the nucleation of HAP and F-HAP on pure ACP, detected a shift in the (PO_4_^3-^) band position to higher level (~2cm^-1^) when F-HAP was nucleated other than HAP. However, EDXS results (Fig. 10) did not support the presence of F-HAP or FAP in NP/II, as no considerable fluoride mass % was recorded [51]. This could be attributed to the low sensitivity of EDXS to fluoride when it exists in small amounts [54].

No evidence of dense mineral layer was detected in SEM micrographs of both NP/III and NP/AS (Fig. 9). Instead, few discrete crystals were deposited on the demineralized enamel surface in NP/III, with no signs of remineralization in NP/AS [7]. Both groups were unable to restore the oriented ordered structure of enamel with a significantly lower I_002_:I_300_ than healthy enamel (Fig. 5B). Minerals deposited in NP/III showed elemental composition of HAP (Fig. 10A) with a Ca/P molar ratio of 1.698. It also showed major peaks of HAP in XRD results (Fig. 5A). These results suggested that the nature of the minerals deposited was HAP. The difference in SEM micrographs of NP/III and NP/AS (Fig. 9) could be attributed to the ability of fluoride incorporated in the remineralization protocol in NP/III to increase the resistance of HAP to pH-cycling.

In peptide-treated groups, continuous dense homogenous layers of nanocrystals that are clumped together in a directional pattern were deposited on the surface in both P/I and P/II (Fig. 8). However, the characteristic elongated nano-rods of native enamel were not detected. On the other hand, in P/III, when enamel surfaces were remineralized with peptide aided only by fluoride (1100 ppm), a discontinuous mineral layer was observed, exposing the demineralized enamel prisms. When the peptide was used alone, depending on the ionic concentration of artificial saliva in P/AS, discrete deposits were detected, instead of forming a dense layer (Fig. 8). All peptide-treated groups displayed the exact XRD major peaks as in healthy enamel (Fig. 5A) with a Ca/P molar ratio (Fig. 10A) close to that of healthy enamel, suggesting that the formed nanocrystallites are considered HAP [19, 21].

The effect of the proper and efficient design of this peptide was profound on both crystal orientation (I_002_:I_300_) and mineral content recovery (ICR%). The peptide was able to enhance crystal growth in the preferred c-axis direction (Fig. 5B) through its hydrophilic C-terminus with a significant enhancement in I_002_:I_300_ of peptide-treated groups in comparison to non-peptide treated groups and demineralized enamel [21]. Additionally, a significant improvement in both Raman intensity of (PO^3-^) peak and, subsequently, the ICR% was detected in peptide-treated groups (Fig. 6C), confirming that the used peptide allowed for ionic interaction and provided a high degree of local supersaturation needed for mineral nucleation [21]. Also, these promising results might be attributed to the repeated daily peptide application that promoted prolonged adsorption of the peptide on active dental lesions [21].

The incorporation of supersaturated remineralizing solutions in the remineralization protocols in P/I, P/II, NP/I and NP/II resulted in a significant increase in their mineral content recovery (ICR%) (Fig. 6C) in comparison to other groups. The difference in ICR% of both P/II and NP/II groups compared to P/I and NP/I groups might be accredited to the inclusion of fluoride in their remineralizing solutions, which stabilized the newly formed crystals against acidic attacks in pH-cycling [19, 21].

Both P/II and P/III showed a significant increase in fluoride mass % (0.488 and 0.474, respectively) compared to all other groups (Fig. 10B), which might highlight the deposition of F-HAP. These results were in agreement with Clift et al .[51] when they investigated fluorine saturation levels in hydroxyapatite crystals using Solid-State NMR. They reported partial substitution of OH^-^ with fluorine in HAP at 0.54 weight %, resulting in HAP transformation into F-HAP [51]. However, Raman spectral analysis suggested the presence of F-HAP on the enamel surface after remineralization in P/II only. It showed a shift in the position of the (PO_4_^3-^) band to a higher value (Fig. 6B) [52, 53].

Furthermore, fluoride worked synergistically with peptide in group P/II, in significantly directing crystals growth in the best c-axis orientation and enhancing the remineralized enamel mineral content compared to other experimental groups. This was in accordance with Ding et al .[32] as they reported a better c-axis orientation of HAP when NaF was used with the peptide for enamel remineralization.

The presence of discrete deposits of HAP in group P/AS compared to NP/AS that showed no signs of remineralization (Figs. 8 and 9), confirmed that the peptide could adsorb and crystalize ions from artificial saliva in an ordered manner. The mineral deposition in P/AS demonstrated the ability of the artificial saliva to aid enamel remineralization when supplemented by peptide treatment. Chu et al .[20] considered saliva as a potential mineral source that is supersaturated with calcium and phosphate ions for enamel remineralization.

Surface microhardness tests have been commonly used to evaluate the degree of enamel demineralization and remineralization in terms of changes in mineral content and crystalline structure [35, 36, 55]. In the present study, the ability of different remineralization protocols to recover healthy enamel microhardness was measured as the percentage of surface microhardness recovery ratio (SMHRR%).

In their study on enamel deformation behaviour under nanoindentation test, Shen et al. [56] stated that enamel deformation is influenced by its anisotropic hierarchical structure, size and orientation of enamel rods and crystal composition. An indentation depth of 2000 nm was used to evaluate the healthy enamel's nanohardness and elastic modulus to allow the indent to extend across multiple rods with different orientations [56]. The principal advantage of using the continuous stiffness method to calculate H and E from the nanoindentation test, is that it enables continuous measurement as a function of indentation depth with only a single load-unload cycle. This advantage is beneficial for measuring the nanomechanical properties of enamel, in which the mechanical properties vary according to the microstructure with indentation depth [57].

None of the suggested treatments was able to 100% recover the microhardness, elastic modulus, or nanohardness of healthy enamel; however, all groups showed significant enhancements when compared to demineralized enamel (Figs 11 and 12). All peptide-treated groups showed significantly higher recovery values of mechanical properties and degree of orientation of treated enamel than their corresponding alternatives in non-peptide-treated groups. This implied that the used peptide promoted remineralization with organized mineral crystal growth, which positively enhanced enamel mechanical properties. In both peptide-treated groups and non-peptide-treated groups, the results of SMHRR%, R_E_% and R_H_% followed the same pattern observed in ICR%, highlighting that the difference in ICR% and, subsequently, the enamel mechanical properties is also considered a function of remineralizing solutions used in different groups. Groups where remineralizing solution II, containing fluoride and supersaturated with calcium and phosphate ions, was used (P/II and NP/II) showed significantly the highest values, followed by solution I (P/I and NP/I) and finally solution III containing 1100 ppm fluoride only (P/III and NP/III).

Although no significant difference was detected in the c-axis orientation between P/I and P/III, P/I showed significantly higher SMHRR%, R_E_% , R_H_% and ICR% than P/III, which confirmed that mechanical properties of enamel were not only affected by crystal orientation but also by mineral content as well. Furthermore, the effect of the favourable c-axis orientation related to the peptide on enamel mechanical properties was confirmed where P/III and P/AS had significantly higher SMHRR%, R_E_% and R_H_% than NP/II and NP/I, respectively. Although no significant difference was detected in ICR% between P/III and NP/II and between P/AS and NP/I, both peptide-treated groups showed significantly higher I_002_:I_300_.

NP/II showed an exceptional enhancement in its SMHRR% when compared to P/AS. This could be attributed to the significantly higher ICR% detected in NP/II and the possibility of F-HAP crystallization as suggested by Raman results (Fig. 6A). Also, the discontinuity and clefts detected in SEM micrographs of P/AS (Fig. 8) would have significantly affected its surface microhardness.

Additionally, a discrepancy was detected while comparing elastic modulus and nanohardness readings of NP/II and P/AS. Although no significant difference was detected between both groups regarding nanohardness, NP/II showed significantly lower elastic modulus value. This finding attracted the attention toward pores detected underneath the remineralized layer in NP/II in a cross-sectional view (Fig. 9). Wei et al. [58] in their study, detected a decrease in elastic modulus of remineralized enamel when the surface layer was relatively loose and detached from the native enamel surface. They also observed a larger zone of permanent deformation when investigated using Nanoscale dynamic mechanical analysis (Nano-DMA )[58]. Therefore, further investigations regarding testing, mapping the deformation behaviour and studying crack behaviour in relation to indentation direction are recommended [56, 58].

Based on the results obtained in the current study, the synergistic application of amelogenin-inspired peptide with calcium phosphate solution and fluoride seems to be a promising approach for enamel biomimetic remineralization. Nevertheless, further research is needed to provide a clinically acceptable delivery system for this combo.

## Conclusion

Despite pH changes, the tested peptide was capable of remineralizing enamel with ordered crystals. Moreover, the supplementary use of calcium phosphate fluoride solution with peptide granted an enhancement in enamel mechanical properties after remineralization.

## Data Availability

The datasets used and/or analysed during the current study are available from the corresponding author on reasonable request.
